# Pirtobrutinib, a highly selective, noncovalent (reversible) BTKi in R/R follicular lymphoma: phase 1/2 BRUIN study

**DOI:** 10.1182/bloodadvances.2024014975

**Published:** 2025-08-25

**Authors:** Nirav N. Shah, Pier Luigi Zinzani, Michael Wang, Sunita D. Nasta, Ewa Lech-Maranda, Yoshiaki Ogawa, Bita Fakhri, Bryone Kuss, Kaname Miyashita, Krish Patel, Catherine C. Coombs, Shuo Ma, Manish R. Patel, Minal A. Barve, Benoit Tessoulin, Anastasios Stathis, Daisuke Ennishi, Daigo Hashimoto, Kensuke Kojima, Andrew D. Zelenetz, Jonathon B. Cohen, Julie M. Vose, Kami J. Maddocks, Talha Munir, Fangfang Sun, Yuanyuan Bian, Minna Balbas, Donald E. Tsai, Paolo Abada, Chan Y. Cheah

**Affiliations:** 1 Division of Hematology and Oncology, Medical College of Wisconsin, Milwaukee, WI; 2 Istituto di Ematologia “Seràgnoli,” IRCCS Azienda Ospedaliero-Universitaria di Bologna, Bologna, Italy; 3 Dipartimento di Scienze Mediche e Chirurgiche, Università di Bologna, Bologna, Italy; 4 Department of Lymphoma & Myeloma, MD Anderson Cancer Center, Houston, TX; 5 Division of Hematology Oncology, Abramson Cancer Center, University of Pennsylvania, Philadelphia, PA; 6 Institute of Hematology and Transfusion Medicine, Warsaw, Poland; 7 Department of Hematology/Oncology, Tokai University School of Medicine, Kanagawa, Japan; 8 Division of Hematology, Stanford University School of Medicine, Stanford, CA; 9 Haematology and Molecular Genetics, Flinders Medical Centre and Flinders University, Bedford Park, SA, Australia; 10 Department of Hematology, National Hospital Organization Kyushu Cancer Center, Fukuoka, Japan; 11 Sarah Cannon Research Institute, Nashville, TN; 12 Division of Hematology/Oncology, University of California Irvine, Irvine, CA; 13 Hematology and Oncology, Robert H. Lurie Comprehensive Cancer Center of Northwestern University, Chicago, IL; 14 Florida Cancer Specialists, Sarasota, FL; 15 Sarah Cannon Research Institute, Nashville, TN; 16 Mary Crowley Cancer Research, Dallas, TX; 17 Nantes University School of Medicine and University Hospital, Nantes, France; 18 New Drugs Development Unit, Oncology Institute of Southern Switzerland, Ente Ospedaliero Cantonale, Bellinzona, Switzerland; 19 Department of Hematology and Oncology, Okayama University Hospital, Okayama, Japan; 20 Department of Hematology, Hokkaido University Hospital, Sapporo, Japan; 21 Department of Hematology, Kochi Medical School Hospital, Nankoku, Japan; 22 Memorial Sloan Kettering Cancer Center, New York, NY; 23 Winship Cancer Institute, Emory University, Atlanta, GA; 24 Division of Oncology and Hematology, University of Nebraska Medical Center, Omaha, NE; 25 Division of Hematology, The Ohio State University Comprehensive Cancer Center, Columbus, OH; 26 Department of Haematology, St James's University Hospital, Leeds, United Kingdom; 27 Eli Lilly and Company, Indianapolis, IN; 28 Linear Clinical Research, Sir Charles Gairdner Hospital and University of Western Australia, Nedlands, WA, Australia

## Abstract

Relapsed/refractory (R/R) follicular lymphoma (FL) is a chronic disease often requiring multiple lines of therapy. Covalent Bruton tyrosine kinase inhibitor (BTKi) monotherapy has resulted in variable response rates, yet patients invariably experience relapse. While newer therapies such as bispecific antibodies and chimeric antigen receptor T-cell (CAR T cell) therapy are available, patient access and eligibility remain challenging. Here, we report the safety and efficacy of pirtobrutinib, a noncovalent (reversible) BTKi monotherapy in a R/R FL cohort from the multicenter phase 1/2 BRUIN study. Key end points included investigator-assessed overall response rate (ORR) per Lugano 2014 criteria, duration of response (DoR), progression-free survival (PFS), overall survival (OS), and safety. Among 48 patients with FL, the median age was 64.5 years (range, 37.0-85.0). Patients had received a median of 3 (range, 1-12) prior lines of therapy. The ORR with pirtobrutinib was 52.1% (95% confidence interval [CI], 37.2-66.7), and median DoR was 10.2 months (95% CI, 3.7-25.7). Median PFS was 5.8 months (95% CI, 3.8-8.1), and median OS was not estimable, with a median follow-up of 35.2 months (interquartile range, 31.1-41.8). The estimated DoR, PFS, and OS rates at 24 months were 33.3% (95% CI, 15.9-51.9), 25.6% (95% CI, 13.9-39.1), and 75.1% (95% CI, 59.5-85.4), respectively. Pirtobrutinib was well tolerated, with 2 patients (4.2%) discontinuing treatment due to adverse events (AEs; 1 treatment-related) and 4 patients (8.3%) having dose reductions due to AEs (all treatment related). Pirtobrutinib showed promising efficacy and was well tolerated in this cohort of patients with heavily pretreated R/R FL, warranting further investigation. This trial was registered at www.clinicaltrials.gov as #NCT03740529.

## Key Points

• Pirtobrutinib demonstrates efficacy in R/R FL, with an ORR of 52.1% and median DoR of 10.2 months.
• Pirtobrutinib has a favorable safety profile in R/R FL, with low rates of dose reduction (8.3%) and discontinuation (4.2%) due to AEs.

## Visual Abstract

**Figure:**
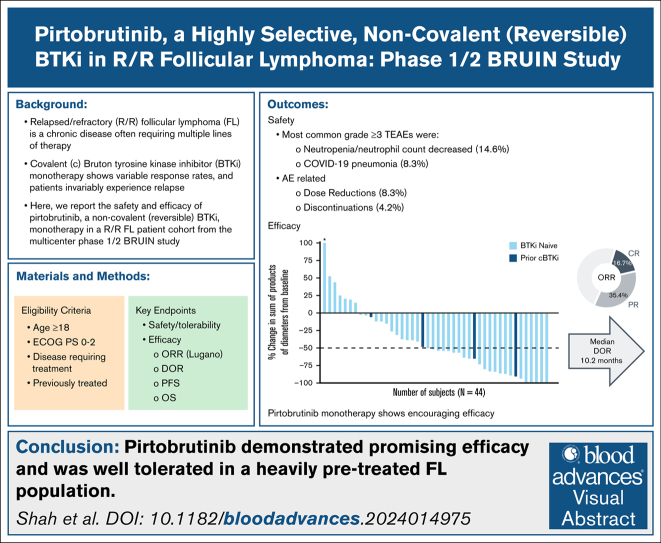

## Introduction

Follicular lymphoma (FL) is the second most common type of non-Hodgkin lymphoma in Western countries, accounting for ∼20% of cases.^1^, ^2^, ^3^ Many patients present with advanced-stage disease at diagnosis.^2^^,^^3^ The disease remains largely incurable, and most patients will have a persistent relapsing course requiring multiple lines of therapy to control disease, including use of chemoimmunotherapy, hematopoietic cell transplantation, bispecific antibody, or cellular therapy. Unfortunately, increasing lines of therapy have been associated with shorter durations of survival,^4^ and there is a need to develop further therapies to improve outcomes.

Covalent Bruton tyrosine kinase inhibitors (cBTKis) have played a significant role in the management of several B-cell malignancies, particularly chronic lymphocytic leukemia (CLL) and mantle cell lymphoma (MCL). Monotherapies using a cBTKi have demonstrated favorable safety profiles, with most side effects being mild or moderate in severity.^5^^,^^6^ However, despite the considerable impact in CLL and MCL, the efficacy of single-agent cBTKis has been limited in patients with relapsed/refractory (R/R) FL, with an overall response rate (ORR) of 20.9% to 37.5% with ibrutinib,^7^^,^^8^ and 36.4% with zanubrutinib.^9^ These monotherapy results have prompted the investigation of combinations with other agents, such as zanubrutinib with the anti-CD20 monoclonal antibody obinutuzumab,^10^ which was ultimately approved for the treatment of FL but is still not curative.^11^^,^^12^

Given the limitations of current covalent inhibitors, a BTK inhibitor with improved efficacy or that could be used after progression after a previous cBTKi would be of high interest. Pirtobrutinib, a highly selective, noncovalent (reversible) BTKi presents favorable oral pharmacology that enables continuous BTK inhibition throughout the once-daily dosing interval, independently of the intrinsic BTK turnover rate.^13^ The selective nature of pirtobrutinib can also reduce off-target inhibition, consequently minimizing adverse events (AEs) while enabling maximal on-target drug coverage.^14^

Pirtobrutinib has demonstrated promising efficacy and tolerability in the phase 1/2 BRUIN study of heavily pretreated patients with poor-prognosis B-cell malignancies, including those that had been treated previously with other cBTKi therapies.^15^^,^^16^ Based on these efficacy and safety results, pirtobrutinib has received approval from the US Food and Drug Administration and the European Medicines Agency for the treatment of adult patients with MCL^17^^,^^18^ and CLL/small lymphocytic lymphoma.^19^^,^^20^ Here, we report the safety and efficacy of pirtobrutinib in a cohort of 48 patients with R/R FL from the phase 1/2 BRUIN study.

## Methods

### Study design

Patient assignment by study phase and B-cell malignancy in the BRUIN phase 1/2 study is represented in supplemental Figure 1. This trial was registered with ClinicalTrials.gov (identifier: NCT03740529).

### Patients

Eligibility criteria for the entire patient cohort enrolled in the BRUIN trial have previously been described in detail.^15^ Briefly, eligible patients were aged ≥18 years, had an Eastern Cooperative Oncology Group performance status score of 0 to 2, had histologically confirmed active FL, and had received at least 1 previous regimen. There was no limit on previous lines of therapy (type or number), and previous treatment with cBTKi was permitted. The FL International Prognostic Index (FLIPI) risk was determined at time of study entry.^21^ Patients with FL were treated in either the dose escalation or expansion phase of the trial. As part of the expansion phase of the study, enrollment of patients with FL beyond 20 patients was permitted if response was observed in at least 20%.

### Outcomes

Efficacy end points for patients with FL included investigator-assessed ORR per Lugano 2014 criteria, duration of response (DoR), progression-free survival (PFS), and overall survival (OS). Disease response assessments were implemented every 8 weeks during the first year, every 12 weeks during the second year, and every 6 months thereafter.

Treatment-emergent adverse events (TEAE) were defined as all AEs reported from the date of the first dose through the last dose date plus 37 days or start of subsequent anticancer therapy, whichever was earlier. Severity of TEAEs was graded by the investigator per the National Cancer Institute Common Terminology for Adverse Events, version 5.0, and attribution of TEAE were assigned by the investigator. The reported AE term is coded using version 26.0 of the Medical Dictionary for Regulatory Activities.

### Statistical analysis

A data cutoff 27 January 2025 was established for all analyses. Descriptive statistics were used to describe patient disposition, demographics, baseline disease characteristics, best response (complete response [CR], partial response [PR], or stable disease), and safety data. ORR was estimated with an exact, 2-sided 95% confidence interval (CI). PFS was measured from the treatment start date until the first date of progression or death, and DoR was measured from the first response date until the first date of progression or death. Patients without progression or death were censored at the last adequate disease assessment, and those who started a subsequent anticancer therapy before progression or death were censored at the last adequate disease assessment before the start of subsequent anticancer therapy. OS was measured from treatment start date until death from any cause, and patients who were alive or lost to follow-up at last contact were censored. Distributions of all time-to-event end points were estimated using the Kaplan-Meier method. All analyses were conducted using SAS version 9.4.

The trial protocol was approved by the institutional review boards overseeing each participating site. The trial was conducted in accordance with the Declaration of Helsinki, good clinical practice guidelines, and local laws. All patients provided written informed consent.

## Results

### Patient baseline characteristics

From October 2019 to June 2022, a total of 48 patients with FL were enrolled and treated with at least 1 dose of pirtobrutinib (Table 1). Patients had a median age of 64.5 years (range, 37.0-85.0), and were predominately male (n = 29 [60.4%]). All patients had an Eastern Cooperative Oncology Group performance status score of 0 (n = 26 [54.2%]) or 1 (n = 22 [45.8%]). The FLIPI risk was low (0-1) in 9 (18.8%) patients, intermediate (2) in 14 (29.2%), high (3-5) in 23 (47.9%), and missing in 2 (4.2%). Patients had a median of 3 (range, 1-12) previous lines of therapy. Patients had received the following treatments: anti-CD20 antibody (100%, n = 48), chemotherapy and an anti-CD20 antibody (n = 43 [89.6%]), phosphoinositide 3-kinase inhibitor (n = 17 [35.4%]), lenalidomide (n = 14 [29.2%]), autologous stem cell transplant (n = 6 [12.5%]), bispecific antibodies (n = 5 [10.4%]), cBTKi (n = 4 [8.3%]), and chimeric antigen receptor (CAR) T-cell therapy (n = 4 [8.3%]). Of the 4 (8.3%) patients who had received a previous cBTKi, 3 discontinued the previous cBTKi due to disease progression (2 patients treated with zanubrutinib, 1 patient treated with ibrutinib) and 1 discontinued for intolerance (zanubrutinib). Among 48 patients with FL, 45 (93.8%) patients received the recommended phase 2 dose of pirtobrutinib (200 mg once daily) as their starting dose, 2 patients (4.2%) received 250 mg and 1 patient [2.1%] received 150 mg once daily.

**Table 1.** **Patient baseline characteristics**

Characteristics	N = 48
Median age (range), y	64.5 (37.0-85.0)
Male, n (%)	29 (60.4)
**ECOG PS**, n (%)	
0	26 (54.2)
1	22 (45.8)
Median number of previous lines of systemic therapy (range)	3 (1-12)
**Previous therapy, n (%)**	
Anti-CD20 antibody	48 (100)
Chemotherapy and anti-CD20 antibody	43 (89.6)
PI3K inhibitor	17 (35.4)
Lenalidomide	14 (29.2)
Autologous stem cell transplant	6 (12.5)
cBTKi	4 (8.3)
CAR T-cells	4 (8.3)
BCL2 inhibitor	3 (6.3)
Other systemic therapy∗	18 (37.5)
**FLIPI risk group, n (%)**	
Low (0-1)	9 (18.8)
Intermediate (2)	14 (29.2)
High (3-5)	22 (45.8)
Missing	3 (6.3)
**Involved nodal sites, n (%)**	
≤4	27 (56.3)
>4	19 (39.6)
Missing	2 (4.2)
**Tumor bulk (cm), n (%)**	
<5	33 (68.8)
≥5	10 (20.8)
No measurable lymph node	5 (10.4)
**Ann Arbor staging, n (%)**	
I/II	8 (16.7)
III/IV	39 (81.3)
Missing	1 (2.1)
**Baseline LDH > ULN, n (%)**	
Yes	14 (29.2)
No	34 (70.8)
**Baseline hemoglobin of <120 g/L, n (%)**	
Yes	14 (29.2)
No	34 (70.8)

Data cutoff of 27 January 2025.

BCL2, B-cell lymphoma 2; ECOG PS, Eastern Cooperative Oncology Group performance status; LDH, lactate dehydrogenase; PI3K, phosphoinositide 3-kinase; ULN, upper limit of normal.

∗ Other systemic therapies include: histone deacetylase inhibitors, radioimmunotherapy, anti-CD74 antibodies, and proteasome inhibitors

### Efficacy

**Figure 1.**
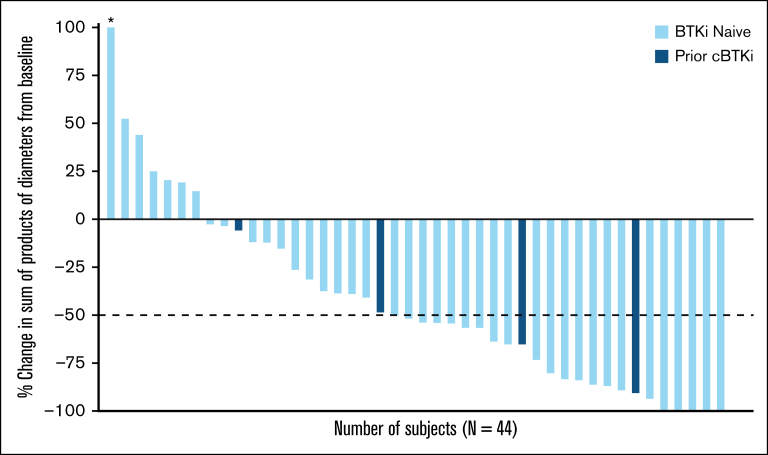
**Pirtobrutinib efficacy in patients with R/R FL.** Best change in sum of product diameters from baseline. Data for 4 patients are not shown in the waterfall plot due to no measurable target lesions identified by computed tomography scan at baseline, discontinuation before first response assessment, or lack of adequate imaging in follow-up. Data cutoff of 27 January 2025. ∗Indicates patient with a >100% increase in sum of products of diameter, with the corresponding change from baseline of 145.1.

The ORR in the overall FL population was 52.1% (95% CI, 37.2-66.7), including 8 (16.7%) CR and 17 (35.4%) PR (Table 2). Among the 4 patients who had received previous cBTKi, 3 achieved PR and 1 had stable disease. As represented in the waterfall plot (Figure 1), among patients with baseline and postbaseline tumor assessment (n = 44), 37 (84.1%) achieved a reduction in sum of product of tumor diameters from baseline, which was similarly observed in the small number of patients with prior cBTKi treatment (n = 4 [100%]).

**Table 2.** **Best overall response among patients with R/R FL**

Best Overall Response	All patients with FL N = 48
ORR∗, % (95% CI)	52.1 (37.2-66.7)
**Best response, n (%)**	
CR	8 (16.7)
PR	17 (35.4)
SD	11 (22.9)
PD	11 (22.9)
NE	1 (2.1)

Data cutoff of 27 January 2025.

CR, complete response; FL, follicular lymphoma; NE, not evaluable; PD, progressive disease; PR, partial response; SD, stable disease

∗ ORR is defined as the number of patients with best response of CR or PR divided by the total number of patients; 1 patient with a best response of NE is included in the denominator.

**Figure 2.**
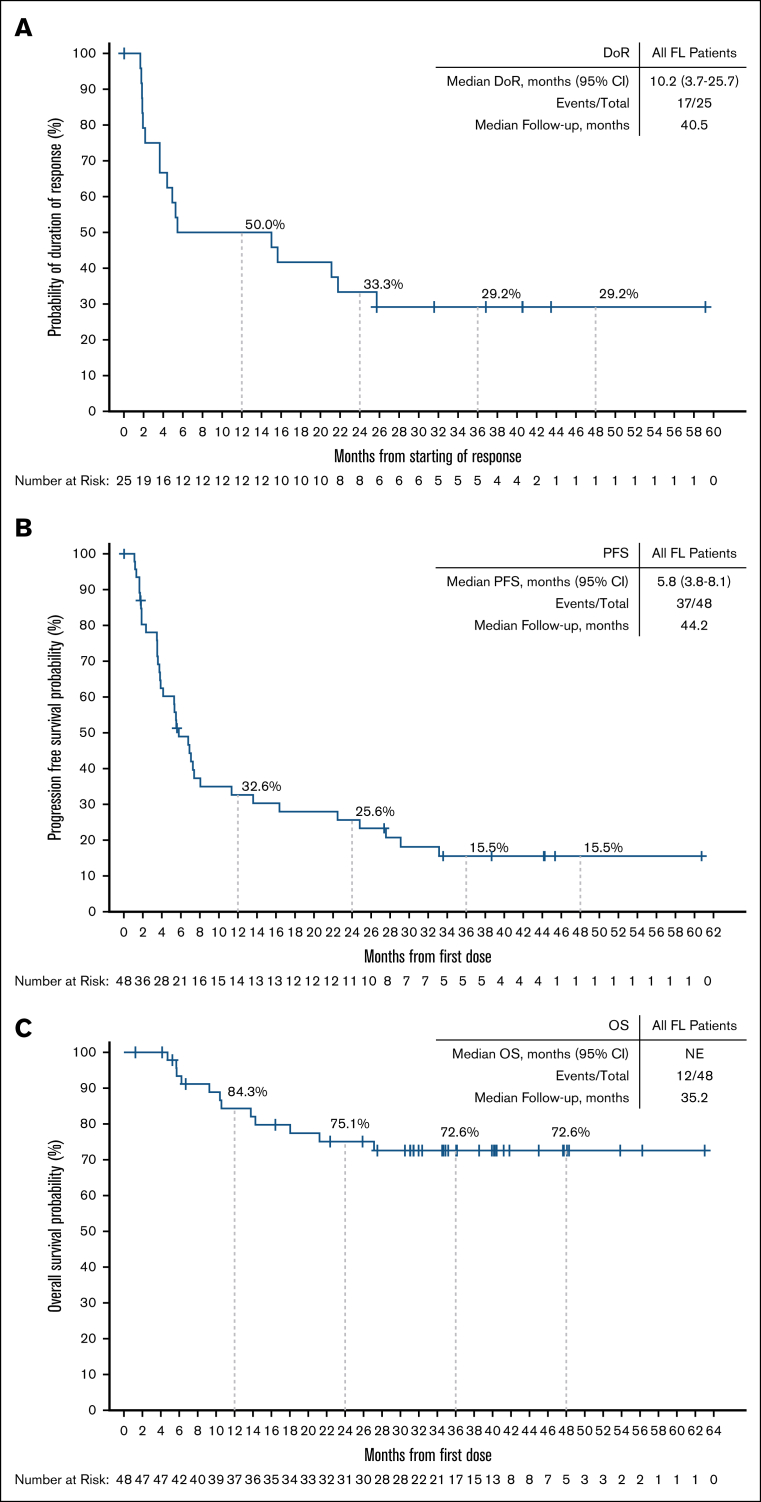
**DoR, PFS, and OS among patients with R/R FL.** Kaplan-Meier curves describing the investigator-assessed DoR (A) and PFS (B) per Lugano 2014 criteria, and OS (C) of all patients with R/R FL enrolled in the BRUIN study. Patients who were alive and without documented progressive disease (DoR, PFS) as of the data analysis cutoff date were censored. Patients who were alive or lost to follow-up (OS) as of the data analysis cutoff date were censored. Data cutoff of 27 January 2025. NE, not estimable.

**Figure 3.**
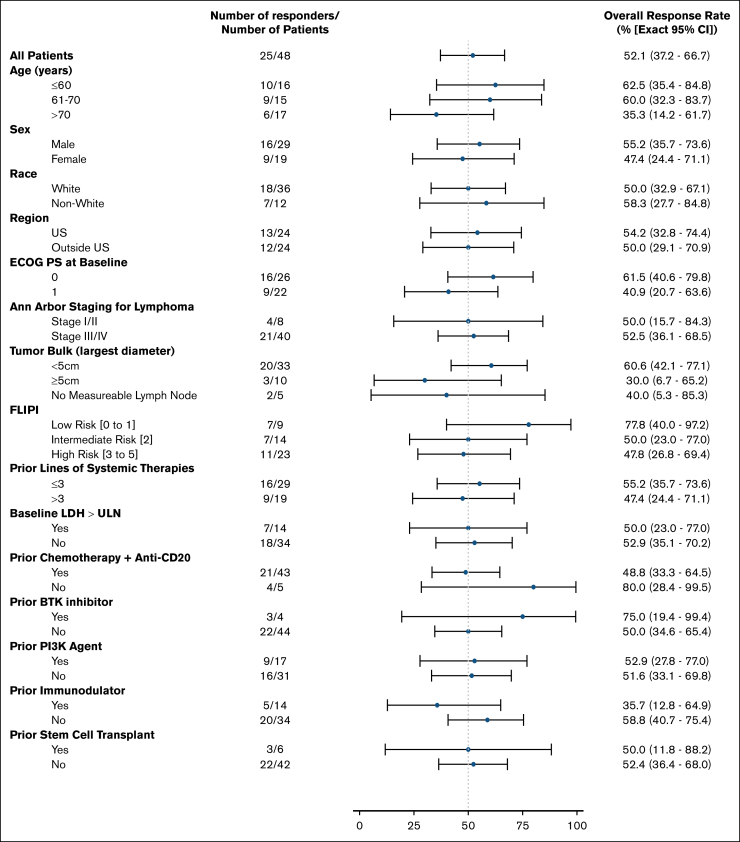
**ORR in patient subgroups.** Subgroup analysis of investigator-assessed ORR per Lugano 2014 criteria. Data cutoff of 27 Jan 2025. BCL2, B-cell lymphoma 2; ECOG PS, Eastern Cooperative Oncology Group performance status; LDH, lactate dehydrogenase; PI3K, phosphoinositide 3-kinase; ULN, upper limit of normal.

**Figure 4.**
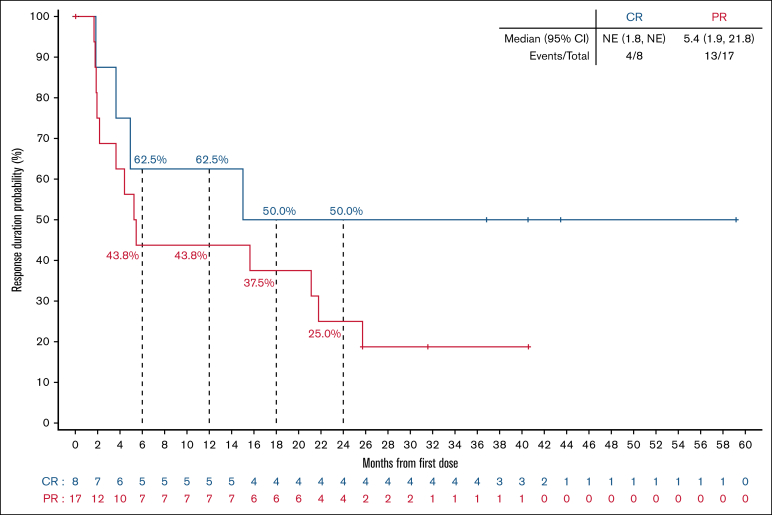
**DoR by best overall response.** Kaplan-Meier curve of investigator-assessed DoR per Lugano 2014 criteria for patients with R/R FL. Patients with CR or PR from first response. NE, not estimable.

Among responding patients, the median time to response was 1.9 months (range, 1.6-17.5), corresponding to the timing of the initial response assessment (supplemental Figure 2). With a median follow-up of 40.5 months (interquartile range, 31.6-43.5), the median DoR was 10.2 months (95% CI, 3.7-25.7), and the 24-month estimated DoR rate was 33.3% (95% CI, 15.9-51.9; Figure 2A). Among 8 patients with best response of CR, 4 (50%) patients had disease progression within the first 18 months after achieving a response, but the remaining 4 patients remain in CR at 36.8, 40.5, 43.5, and 59.2 months, respectively (Figure 3). In the overall FL population, the median PFS was 5.8 months (95% CI, 3.8-8.1), and the 24-month estimated PFS rate was 25.6% (95% CI, 13.9-39.1; Figure 2B). Among 17 patients who had not responded to their most recent previous therapy, the median PFS was 4.1 months (95% CI, 3.5-7.3); for the 26 patients who had responded to their most recent prior therapy, the median PFS was 6.8 months (95% CI, 2.3-27.6; Figure 4). With a median follow-up of 35.2 months (interquartile range, 31.1-41.8), the median OS in the overall FL population was not reached, and the 24-month estimated OS rate was 75.1% (95% CI, 59.5-85.4; Figure 2C).

**Figure 5.**
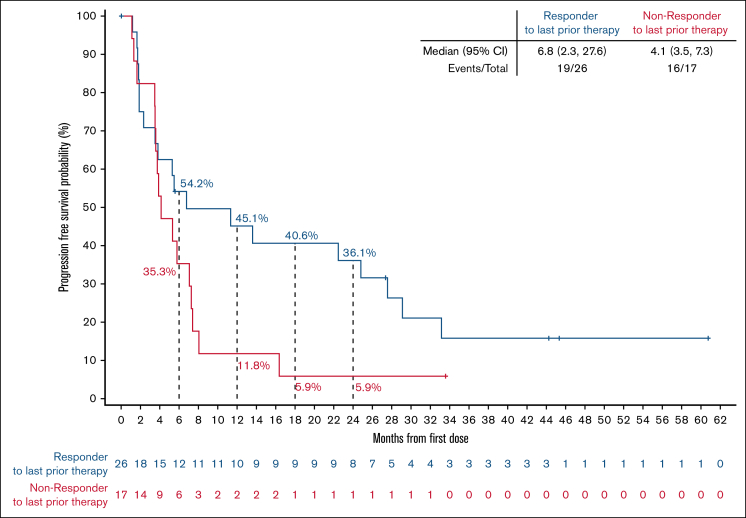
**PFS by response status to most recent previous therapy.** PFS by response status to most recent previous therapy. Data cutoff of 27 January 2025. NE, not estimable.

Focusing on efficacy results by FLIPI risk, the ORR was 77.8% (95% CI, 40.0-97.2) in the low-risk subgroup, 50.0% (95% CI, 23.0-77.0) in the intermediate-risk subgroup, and 47.8% (95% CI, 26.8-69.4) in the high-risk subgroup (Figure 5). The DoR by FLIPI risk is presented in supplemental Figure 3A. The median PFS assessed by FLIPI risk was 8.1 months (95% CI, 3.5-27.6) for the low-risk group, 6.9 months (95% CI, 3.5 to not estimable) for the intermediate-risk group, and 5.3 months (95% CI, 2.3-7.3) for the high-risk group (supplemental Figure 3B), with PFS estimates at 24 months of 22.2% (95% CI, 3.4-51.3), 35.2 (95% CI, 11.2-60.7), and 22.9% (95% CI, 8.4-41.7), respectively.

### Safety

The median time on treatment was 7.6 months (range, 0.6-63.0), with 8 (16.7%) patients still receiving pirtobrutinib at the time of the data cutoff. TEAEs were experienced by 46 (95.8%) patients with FL. The most frequent TEAEs, treatment-related AEs (TRAE), and AEs of interest are shown in Table 3. The most common TEAEs, regardless of attribution, were diarrhea (n = 14 [29.2%]), fatigue (n = 12 [25.0%,]), and nausea (n = 11 [22.9%]). Grade ≥3 TEAEs were experienced by 24 (50.0%) patients. Most common grade ≥3 TEAEs were neutropenia/decreased neutrophil count (n = 7 [14.6%]) and COVID-19 pneumonia (n = 4 [8.3%]). Grade ≥3 TEAE of infections occurred in 9 (18.8%) patients. There was 1 grade 5 (death) TEAE reported (COVID-19 pneumonia), which was considered unrelated to pirtobrutinib treatment by the investigator.

**Table 3.** **Any-grade and grade ≥3 TEAEs occurring in 15% or more of all patients with R/R FL, and TRAEs**

Preferred Term	TEAEs in patients with FL (N = 48)			
	All-cause AEs (≥15%), %		TRAEs, %	
**AE**	**Any grade**	**Grade ≥3**	**Any grade**	**Grade ≥3**
Diarrhea	29.2	2.1	8.3	0
Fatigue	25.0	4.2	14.6	0
Nausea	22.9	2.1	12.5	2.1
Arthralgia	18.8	0	8.3	0
Back pain	18.8	0	0	0
Neutropenia∗	16.7	14.6	10.4	8.3
**AEs of interest**†	**Any grade**	**Grade ≥3**	**Any grade**	**Grade ≥3**
Infections‡	56.3	18.8	12.5	0
Rash§	14.6	2.1	8.3	2.1
Bruising‖	10.4	0	2.1	0
Hemorrhage¶	6.3	0	2.1	0
Hypertension	6.3	2.1	0	0
Atrial fibrillation/flutter#	2.1	0	2.1	0

Data cutoff of 27 January 2025.

∗ Aggregate of neutropenia and neutrophil count decreased.

† AEs of interest are those that were previously associated with cBTKis.

‡ Aggregate of all preferred terms indicating infection and including COVID-19.

§ Aggregate of all preferred terms indicating rash.

‖ Aggregate of contusion, eye contusion, and increased tendency to bruise.

¶ Aggregate of all preferred terms including hemorrhage or hematoma.

# Aggregate of atrial fibrillation and atrial flutter.

Four patients (8.3%) required dose reductions due to TEAEs, all considered treatment-related. Only 2 (4.2%) patients discontinued pirtobrutinib due to TEAE; 1 patient with a treatment-related rash, and 1 patient with hemothorax determined as unrelated to treatment.

TRAEs of any grade were reported in 29 (60.4%) patients, of which 8 (16.7%) were grade ≥3. The most common TRAEs of any grade reported in this population were fatigue (n = 7 [14.6%]), nausea (n = 6 [12.5%]), neutropenia/decreased neutrophil count (n = 5 [10.4%]), and arthralgia and diarrhea (n = 4 for each [8.3%]). Neutropenia/decreased neutrophil count (n = 4 [8.3%]) was the most common grade ≥3 TRAE.

Of the AEs commonly associated with cBTKi therapy, rash occurred in 7 (14.6%) patients, with all but 1 of low grade (grade 1-2); TEAEs of hemorrhage/hematoma (n = 3 [6.3%], all low-grade), hypertension (n = 3 [6.3%], 2 low-grade), and atrial fibrillation/flutter (n = 1 [2.1%], low-grade) were infrequent.

## Discussion

In the phase 1/2 BRUIN trial, we report the results of pirtobrutinib treatment in a cohort of patients with R/R FL. Pirtobrutinib demonstrated efficacy with a favorable safety profile in this cohort of patients with a median of 3 lines of previous therapy, including patients who received previous chemoimmunotherapy as well as a small number of patients who received prior cBTKi treatment. Treatment with pirtobrutinib in R/R FL resulted in an ORR of 52.1% (95% CI, 37.2-66.7), including 8 (16.7%) CR and 17 (35.4%) PR, demonstrating the efficacy of pirtobrutinib in this population. Notably, 4 of 8 patients with CR have ongoing responses 3 to 5 years after first response. Furthermore, consistent ORR values were observed in high-risk groups including those with intermediate (50.0% [95% CI, 23.0-77.0]) and high-risk FLIPI (47.8% [95% CI, 26.8-69.4]; Figure 3). Although cross-trial comparisons are limited and should be interpreted with caution, the ORR presented here are comparable with ORRs reported with cBTKi monotherapies (ibrutinib: 20.9% ORR [95% CI, 13.7-29.7], n = 110^8^; and 37.5% [95% CI, 22.7-54.2], n = 40^7^; acalabrutinib: 33.3% ORR [95% CI, 9.9-65.1], n = 12^22^; and zanubrutinib: 36.4% ORR [95% CI, 20.4-54.9], n = 33^9^). The addition of obinutuzumab to zanubrutinib resulted in improved clinical benefits (ORR, 69%-72%).^10^^,^^23^ However, this combination may come at a cost of increased infection risk.^24^ This combination treatment ultimately received approval by the European Commission and the United States Food and Drug Administration for the treatment of R/R FL.^11^^,^^12^

Currently, the treatment landscape recommendations for R/R FL encompass a variety of approaches, including chemoimmunotherapy; lenalidomide + rituximab; bispecific antibodies; anti-CD19 CAR T-cell therapy; tazemetostat; zanubrutinib + obinutuzumab; and, in selected cases, allogeneic hematopoietic cell transplantation.^25^ In the relapsed setting, with the exception of allogeneic transplant therapy, the available standard therapies are generally not considered curable,^4^^,^^26^ and there remains a critical need for additional effective treatment options for patients with R/R FL. For those patients with FL in their first relapse, chemotherapy or lenalidomide are often used in combination with anti-CD20 or anti-CD19 antibody and have been shown to be highly effective.^27^ Various studies have shown R-CHOP (rituximab, cyclophosphamide, doxorubicin, Oncovin [vincristine], and prednisolone), bendamustine and rituximab (BR), and rituximab with cyclophosphamide, vincristine and prednisone (R-CVP) to be reasonable options in relapsed disease and, similarly, the combination of lenalidomide and rituximab in patients with R/R FL resulted in a reported an ORR of 80% and median DoR of 36.6 months (95% CI, 24.9 to not reached).^28^ For those who require additional therapy, new therapeutic strategies such CAR T-cell therapies, bispecific antibodies, epigenetic inhibitors, and BTKis have been studied for the treatment of R/R FL. However, as of yet, none of these new therapies have proven to be curative and, in many cases, can be challenging to implement. The experience with cBTKis has already been discussed earlier, with the combination of zanubrutinib + obinutuzumab garnering approval in this setting. The CAR T-cell therapies lisocabtagene maraleucel, tisagenlecleucel, and axicabtagene are approved for use in R/R FL, with high ORRs of 86% to 97%, with good durability.^29^, ^30^, ^31^ Despite this promising efficacy, CAR T cells can be logistically challenging with significant toxicities, and unfortunately, many patients are not candidates for this complex therapy. Treatment with mosunetuzumab or epcoritamab, T-cell engaging bispecific antibodies, similarly have shown high ORRs of 77.8% to 82% with durability but can also be associated with unique toxicities like cytokine release syndrome, and rarely immune effector cell–associated neurotoxicity syndrome.^32^^,^^33^ Tazemetostat, an enhancer of zeste homolog 2 inhibitor, is approved by the US Food and Drug Administration as a third-line treatment for patients with R/R FL, and has resulted in an ORR ranging from 35% to 69%, and durability of responses approximating a year, but again does not appear to provide cure.^34^

The mechanisms by which BTK helps drive FL progression are not well understood, much less the mechanisms of acquired resistance to BTKis in this disease.^35^ Mutation analysis of patients with FL who did not respond or have developed resistance to ibrutinib notably did not commonly find mutations in BTK, but rather other genes such as *TP53*, *CARD11*, *EP400*, *ATP6AP1*, *ARID1A*, *SOCS1*, and *TBL1XR1*.^7^^,^^36^ Although numbers are limited, pirtobrutinib showed benefit in 3 of 4 patients that had received previous cBTKi treatment, demonstrating that the BTK pathway can remain an important driver in cBTKi-relapsed FL and pirtobrutinib has activity in this population. While tumor sequencing was not performed in this study of patients with FL, pirtobrutinib has demonstrated broad efficacy in other patients with B-cell malignancies that had progressed in prior BTKi treatment, independently of the presence or absence of mutations known to confer resistance to cBTKis.^15^

Importantly, pirtobrutinib was well tolerated with infrequent reports of AEs associated with BTKi such as rash, high-grade bruising, arthralgia, or major hemorrhage. The safety results in the R/R FL population of pirtobrutinib suggest long-term tolerability with low rates of discontinuations due to AEs (n = 2 [4.2%]). Overall, the safety of pirtobrutinib in patients with R/R FL was consistent with that seen in the larger population of patients treated with pirtobrutinib with diverse B-cell malignancies.^13^ This favorable tolerability profile puts forward pirtobrutinib as a well-tolerated therapeutic option for patients with R/R FL, especially among those who failed previous cBTKi therapy. Considering the efficacy results and the safety profile with pirtobrutinib monotherapy, further evaluation as monotherapy or in combination in the R/R FL population appears warranted. As an example, there is an ongoing phase 1 trial of pirtobrutinib in combination with CAR T-cell therapy in R/R B-cell malignancies, including FL (ClinicalTrials.gov identifier: NCT05990465). In addition, a phase 2 study of pirtobrutinib in combination with bispecific antibody mosunetuzumab in patients with R/R FL is planned (ClinicalTrials.gov identifier: NCT06948786; PROMOTE-FL). These studies may contribute to understanding the potential benefits of pirtobrutinib as a combination partner in R/R FL.

This trial presents some limitations, including the limited number of patients with R/R FL, which affects the in-depth investigation in FL subgroups. Additionally, the lack of comprehensive molecular analysis in FL subgroups limits the possible identification of FL genetic signatures and their potential impact in treatment outcomes. Other limitations include that the BRUIN clinical trial is an open-label, single-arm study, and formal comparison with other available therapies was not possible.

### Conclusion

In summary, pirtobrutinib, a noncovalent (reversible) BTKi, showed promising efficacy and safety as a single agent in a R/R FL cohort. Pirtobrutinib was well-tolerated, with low rates of cBTKi-associated AEs and discontinuations due to AEs. These findings highlight the potential for pirtobrutinib as a clinically meaningful addition to available therapies for patients with R/R FL.

Conflict-of-interest disclosure: N.N.S. reports participation on advisory boards and/or consultancy for Gilead-Kite, Bristol Myers Squibb (BMS)–Juno Therapeutics, Miltenyi Biomedicine, Eli Lilly, Incyte, AbbVie, Cargo, BeiGene, Kite, Allogene, AstraZeneca, BMS, and Galapagos; reports research funding, travel support, and honoraria from Eli Lilly, Genentech, and Miltenyi Biomedicine; and served on the scientific advisory board for Tundra Therapeutics. P.L.Z. reports consulting or advisory role for MSD, Eusa Pharma and Novartis; reports speakers bureau role for Celltrion, Gilead, Janssen-Cilag, BMS, Servier, MSD, AstraZeneca, Takeda, Roche, Eusa Pharma, Kyowa Kirin, Novartis, Incyte and BeiGene; and is a member of an entity's board of directors or advisory committees for Secura Bio, Celltrion, Gilead, Janssen-Cilag, BMS, Servier, Sandoz, MSD, AstraZeneca, Takeda, Roche, Eusa Pharma, Kyowa Kirin, Novartis, ADC Therapeutics, Incyte, and BeiGene. M.W. reports consultancy fees from Acerta Pharma, AstraZeneca, BMS, Boxer Capital, InnoCare, Janssen, Kite Pharma, Eli Lilly, Merck, Physicians Education Resources (PER), Pfizer, and Oncternal; reports research funding from AbbVie, Acerta Pharma, AstraZeneca, Bantam Pharma, BeiGene, BioInvent, Celgene, Genmab, Genentech, Innocare, Janssen, Juno Therapeutics, Kite Pharma, Eli Lilly, Loxo Oncology, Molecular Templates, Nurix Therapeutics, Oncternal, Pharmacyclics, VelosBio, and Vincerx; and reports honoraria from AstraZeneca, BeiGene, BMS, Cahon, Editorial Medica AWWE SA, Instituto Scientifico Romagnolo, Janssen, Kite Pharma, Mayo Clinic, MJH Life Sciences, Merck, MSC National Research Institute of Oncology, PER, Plexus Communications, Studio ER Congressi, South African Clinical Hematology Society, and Medscape/WebMD. S.D.N. reports honoraria from ADC Therapeutics and Accrotech; served as a member on an entity's board of directors or advisory committees for Merck data safety monitoring committee; and reports research funding from Ono Pharmaceutical, Millennium Takeda, Loxo/Eli Lilly, Genentech/Roche, Raphael, and Pharmacyclics. Y.O. reports research funding from Janssen Pharmaceuticals and IQVIA. B.F. reports consultancy for AbbVie, ADC Therapeutics, AstraZeneca, BeiGene, BMS/Juno Therapeutics, Genentech, Genmab/AbbVie, Loxo Oncology, and Pharmacyclics; reports funding (research or clinical trial or other support) from AbbVie, Genentech, Genmab/AbbVie, and Loxo Oncology; and served as a member on an entity’s board of directors, speakers bureau, or advisory committees for AbbVie, ADC Therapeutics, AstraZeneca, BeiGene, BMS/Juno Therapeutics, Genentech, Genmab/AbbVie, Loxo Oncology, and Pharmacyclics. K.P. reports consulting for, or advisory role with, AstraZeneca, Genentech, BeiGene, Pharmacyclics, BMS/Celgene/Juno Therapeutics, MorphoSys, Kite (a Gilead company), TG Therapeutics, Loxo/Eli Lilly, AbbVie, Seagen, Epizyme, ADC Therapeutics, Caribou Biosciences, Xencor, and Fate Therapeutics; reports speakers bureau participation with AstraZeneca, BMS/Celgene, Kite (a Gilead company), and TG Therapeutics; and reports research funding from AstraZeneca (to institution), Xencor (to institution), Pharmacyclics (to institution), Curis (to institution), BMS (to institution), Celgene (to institution), MEI Pharma (to institution), Trillium Therapeutics (to institution), Kite/Gilead (to institution), Roche/Genentech (to institution), Fate Therapeutics (to institution), Takeda (to institution), Epizyme (to institution), Aptevo Therapeutics (to institution), Nurix (to institution), Loxo/Lilly (to institution), and Pfizer (to institution). C.C.C. reports having received honoraria/served as a consultant for AbbVie, Allogene, AstraZeneca, BeiGene, Genentech, Janssen, Lilly, MEI Pharma, MingSight, Octapharma, and TG Therapeutics; served on speakers bureaus for AbbVie, AstraZeneca, BeiGene, Genentech, and Lilly; holds stock in Bluebird Bio, Geron, and Pfizer; and has received research funding (paid to the research institution) from AbbVie, Carna Biosciences, and Lilly. S.M. reports honoraria from National Comprehensive Cancer Network (NCCN), Clinical Care Options, Curio Science, OncLive/MJH Life Sciences, and Research to Practice; reports consulting or advisory role with AbbVie, AstraZeneca, BeiGene, BMS, Genentech, and Janssen Pharmaceuticals; served on speakers bureau for AstraZeneca, Eli Lilly–Loxo Oncology, and BeiGene; and reports research funding from AbbVie, AstraZeneca, BeiGene, Carna Biosciences, Juno Therapeutics–BMS, Janssen Pharmaceuticals, and Eli Lilly–Loxo Oncology. M.R.P. reports consulting fees from Kura, Accutar Biotech, and Mitsubishi; reports participation on a data safety monitoring board or advisory board with Lema, Nurix, Daiichi, Kura, and Janssen; reports leadership with ION Pharma; received honoraria from Janssen Oncology; received research funding from Loxo (to institution); and participated in a consulting or advisory role with Olema Pharmaceuticals, Daiichi Sankyo–UCB Japan, Kura Oncology, Accutar Biotech, and Kura. B.T. reports honoraria from Kite/Gilead and AbbVie; and received funds for travel and accommodation from Kite/Gilead and AbbVie. A.S. reports institutional funding for clinical trials from AbbVie, ADC Therapeutics, Amgen, AstraZeneca, Bayer, BMS, Cellestia, Debiopharm, Incyte, Loxo Oncology, Merck MSD, Novartis, Pfizer, Philogen, Prelude Therapeutics, and Roche; reports consultant/expert testimony/advisory board role (institutional) with Debiopharm, Janssen, AstraZeneca, Incyte, Eli Lilly, Novartis, Roche, and Loxo Oncology; reports (personal) funding from Incyte; and received a travel grant from Incyte and AstraZeneca. D.E. reports honoraria from Kyowa Hakko Kirin Co Ltd, BMS, Ono Pharmaceutical, Janssen, Incyte, Eisai, Chugai, and Nippon Shinyaku Co; and research funding from Eisai, Chugai, and Nippon Shinyaku Co. D.H. reports honoraria from Ono Pharmaceutical, Janssen Pharma, Kyowa Kirin, Chugai Pharmaceutical, Daiichi Sankyo Inc, and Astellas Pharma; and reports patents and royalties from LUCA Science. K.K. reports honoraria from Janssen Pharmaceutical, AstraZeneca, and AbbVie; and reports research funding from Asahi Kasei Pharma Corporation, Kyowa Kirin Co Ltd, Mochida Pharmaceutical Co, Daiichi Sankyo Co Ltd, Eisai Co Ltd, Nippon Kayaku Co, Ltd, Sumitomo Pharma Co Ltd, and Chugai Pharmaceutical Co Ltd. A.D.Z. reports honoraria from NCCN, Curio Science, OncLive/MJH Life Sciences; reports consulting or advisory role with Genentech/Roche, Celgene, AstraZeneca, Dava Oncology, BeiGene, MEI Pharma, Kite (a Gilead company), Juno Therapeutics/Celgene/BMS, Sandoz, and Ono Pharmaceutical; received research funding from Genentech/Roche, MEI Pharma, BeiGene, and AbbVie (to institution); and received travel and accommodation expenses from Kite (a Gilead company), NCCN, and BeiGene. J.B.C. reports funding (research or clinical trial or other support) from Loxo Oncology, Takeda, Novartis, BMS/Celgene, Genentech, AstraZeneca, BioInvent, and Lam Therapeutics; and received consulting fees from Loxo Oncology, BeiGene, Janssen, AbbVie, AstraZeneca, ADCT, and Kite. J.M.V. reports consultancy for AbbVie and MEI Pharma; and received research funding from Eli Lilly and Company, Epizyme, Kite, Loxo, and Novartis. K.J.M. received consulting fees from AbbVie, ADC, AstraZeneca, BMS, Caribou, Eli Lilly, Genentech, Genmab, Incyte, Janssen, Kite, Merck, MorphoSys, and Pharmacyclics. T.M. reports research grant funding from Janssen, AbbVie, and Pharmacyclics; consulted for Alexion, AstraZeneca, BeiGene, Janssen, MorphoSys, Roche, Sunesis, Lilly, and Sobi; and reports honoraria/travel support from AbbVie, AstraZeneca, BeiGene, Gilead, Janssen, Novartis/GlaxoSmithKline, Pharmacyclics, Roche, Sobi, and Alexion. F.S., Y.B., M.B., D.E.T., and P.A. are employees of, and equity holders in, Eli Lilly. C.Y.C. reports consulting/advisory role with, and/or honoraria from, Roche, Janssen, Gilead, AstraZeneca, Lilly, BeiGene, Menarini, Dizal, AbbVie, Genmab, Sobi, and BMS; reports research funding from BMS, Roche, AbbVie, MSD, and Lilly; and received travel support from Eli Lilly and BeiGene. The remaining authors declare no competing financial interests.
