# Supplementary material for: Pirtobrutinib, a highly selective, noncovalent (reversible) BTKi in R/R follicular lymphoma: phase 1/2 BRUIN study
Source: Blood Adv. 2025 Aug 25;9(23):5954–64. doi: 10.1182/bloodadvances.2024014975 (PMC12686794; doi:10.1182/bloodadvances.2024014975)
Supplement: Supplemental Figures [file BLOODA_ADV-2024-014975-mmc1.docx]

**Supplemental Figures**

**
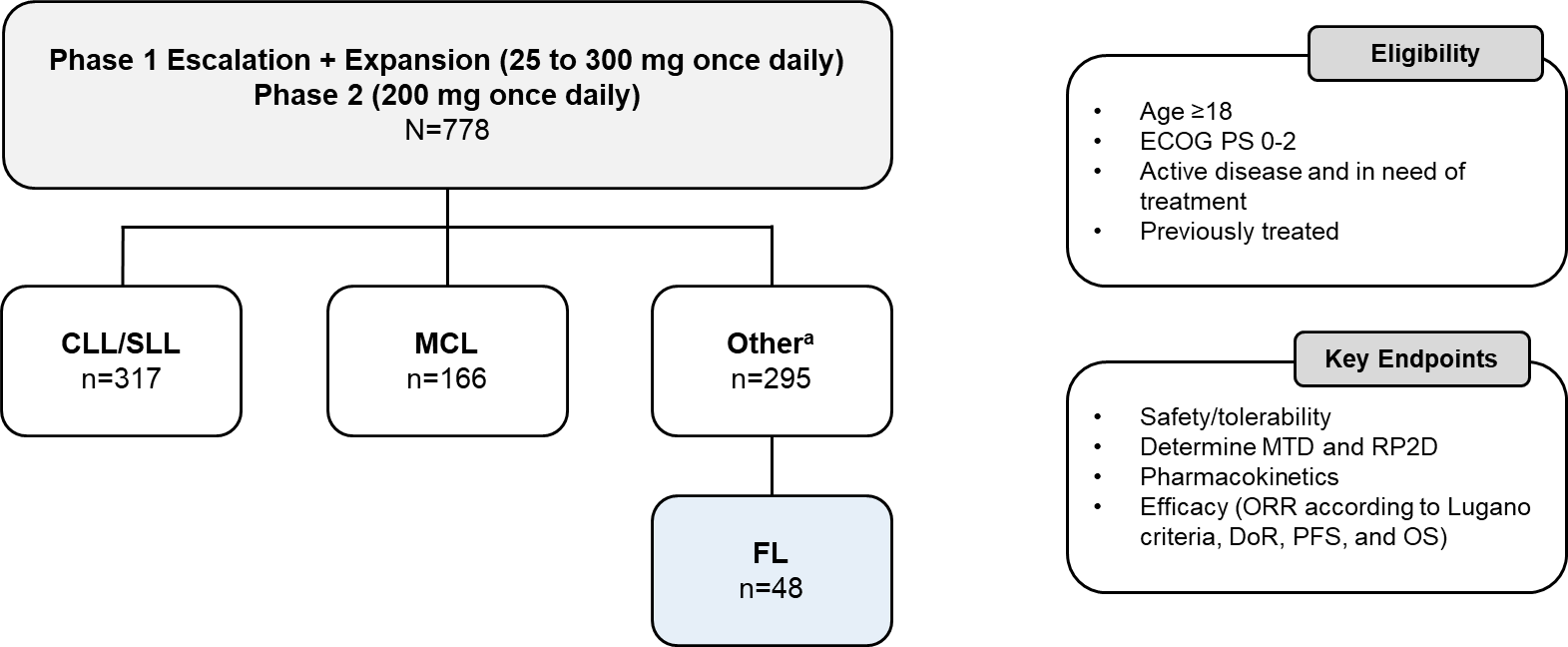
**

**Figure S1 – Phase 1/2 BRUIN Study: Design, Eligibility, and Enrollment.**

Study schema of the Phase 1/2 BRUIN trial with key eligibility criteria and endpoints. Data cutoff of 27 Jan 2025.

^a^Other includes Richter transformation, marginal zone lymphoma, Waldenström macroglobulinemia, hairy cell leukemia, B-cell prolymphocytic leukemia, primary central nervous system lymphoma, and other transformations.

Abbreviations: CLL, chronic lymphocytic leukemia; DoR, duration of response; ECOG PS, Eastern Cooperative Oncology Group performance status; FL, Follicular Lymphoma; MCL, mantle cell lymphoma; MTD, maximum tolerated dose; ORR, overall response rate; OS, overall survival; PFS, progression free survival; RP2D, recommended phase 2 dose; SLL, small lymphocytic lymphoma.

**
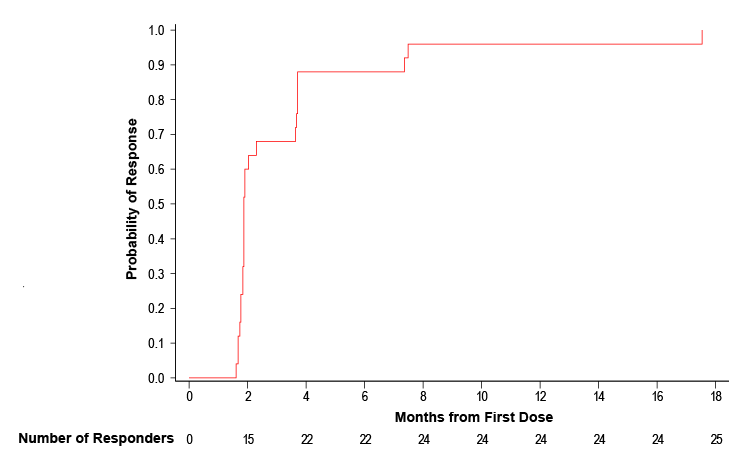
**

**Figure S2 - Time to first response.**

Data cutoff of 27 Jan 2025.


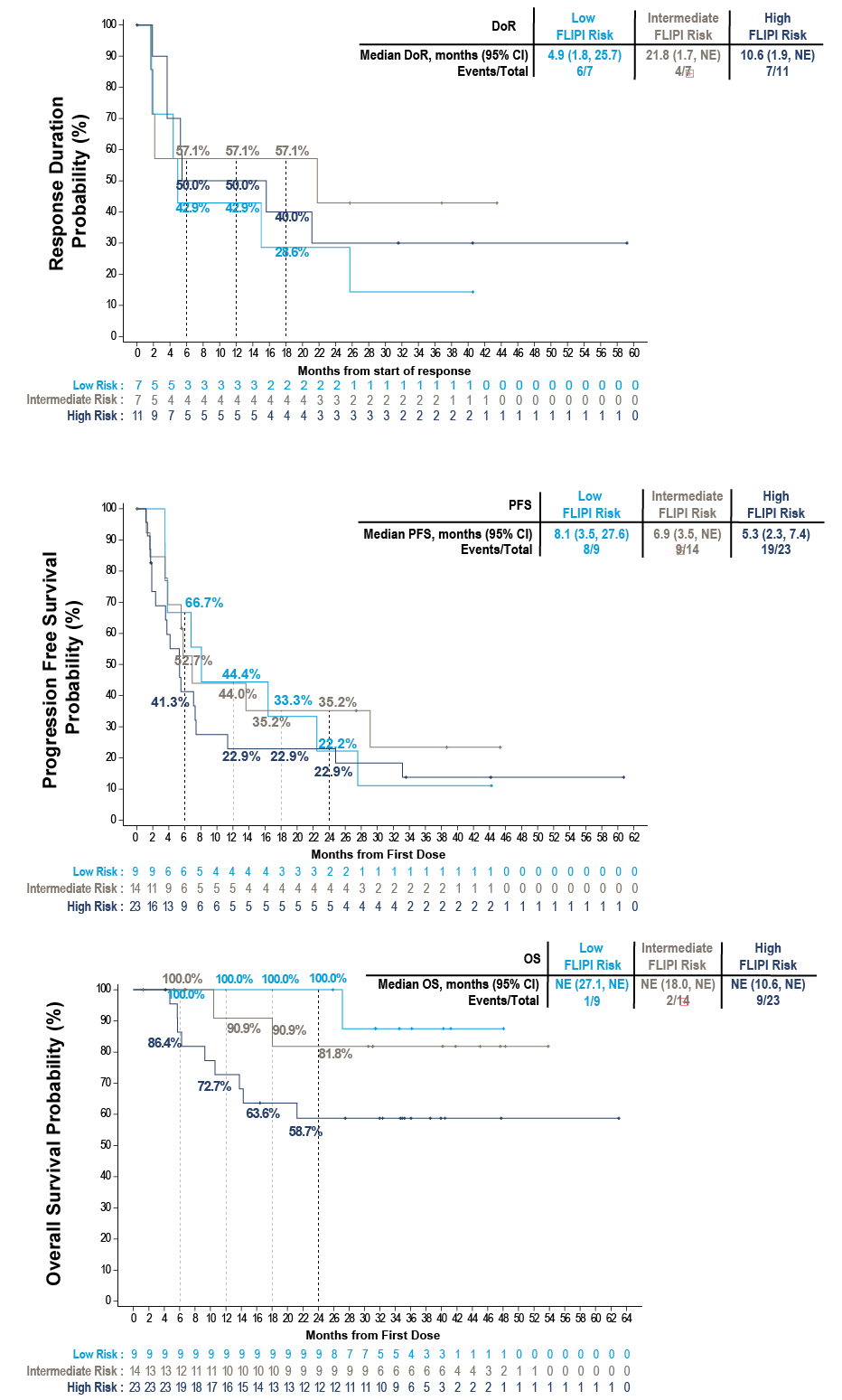


**Figure S3 - DOR, PFS and OS among patients with R/R FL by FLIPI risk.**

Kaplan-Meier curves describing the DOR (a), PFS (b), and OS (c) of all R/R FL patients by FLIPI risk assessment. Data cutoff of 27 Jan 2025.

Abbreviations: CI, confidence interval; DoR, duration of response; FL, Follicular Lymphoma; FLIPI, Follicular Lymphoma International Prognostic Index; NE, not estimable; OS, overall survival; PFS, progression free survival.
